# The stability of soil organic carbon across topographies in a tropical rainforest

**DOI:** 10.7717/peerj.12057

**Published:** 2021-08-27

**Authors:** Yamin Jiang, Huai Yang, Qiu Yang, Wenjie Liu, Zhaolei Li, Wei Mao, Xu Wang, Zhenghong Tan

**Affiliations:** 1 Hainan University, College of Ecology and Environment, Haikou, China; 2 International Center for Bamboo and Rattan, BeiJing, China; 3 Chinese Academy of Forestry, Jianfengling National Key Field Research Station for Tropical Forest Ecosystem, Research Institute of Tropical Forestry, Hainan, China; 4 Northern Arizona University, Center for Ecosystem Science and Society, Flagstaff, AZ, USA

**Keywords:** Soil organic carbon stability, Topography, Tropical forest, 13C CPMAS/NMR, Path analysis

## Abstract

Mechanisms of soil organic carbon (SOC) stability are still unclear in forest ecosystems. In order to unveil the influences of topography on the SOC stability, a 60ha dynamic plot of a tropical montane rainforest was selected in Jianfengling, in Hainan Island, China and soil was sampled from 60 quadrats. The chemical fractions of the SOC were detected with 13C CPMAS/NMR and path analyses explore the mechanisms of SOC stability in different topographies. The chemical fractions of the SOC comprised alkyl carbon > O-alkyl carbon > carboxyl carbon > aromatic carbon. The decomposition index (DI) values were greater than 1 in the different topographies, with an average DI value was 1.29, which indicated that the SOC in the study area was stable. Flat and top areas (together named RF) had more favorable nutrients and silt contents compared with steep and slight steep areas (together named RS). The influencing factors of SOC stability varied across the topographies, where SOC, soil moisture (SM) and ammoniacal nitrogen (NH_4_^+^-N, AN) were the main influencing factors in the RF, while SM and AN were the main factors in the RS. Greater SOC and AN strengthened the SOC stability, while higher soil moisture lowered SOC stability. The inertia index was higher in the RS than the RF areas, indicating that local topography significantly affects SOC content and SOC stability by changing soil environmental factors. Topography cannot be neglected in considering SOC stability and future C budgets.

## Introduction

The soil organic carbon (SOC) of forests plays an important role in the global carbon cycle (Pan et al., 2011). Changes in SOC storage can significantly impact the global carbon cycle and climate change (Stockmann et al., 2013). SOC stability is the ability of soil organic matter to resist disturbance and maintain its original carbon levels under the present conditions (Wiesmeier et al., 2014). High SOC stability would be beneficial for the accumulation of SOC (Schmidt et al., 2011). The SOC stabilisation mechanisms that are commonly accepted include chemical stabilisation, physical stabilisation, and biochemical stabilisation (Christensen, 1996; Fontaine et al., 2007; Six et al., 2002). In terms of chemical stabilisation, SOC consists of chemical fractions of different stability and this has long been considered as the principal mechanism of SOC stability (Yang et al., 2020). The relationship between soil structure and the ability of soil to stabilize soil organic matter indicating that physical protection is a key element in SOC stability (Six et al., 2002). Studies on the protection of soil C by soil texture, such as silt and clay, had long been well established (Hassink, 1997). Solid 13CNMR technology is a method for dissecting SOC chemical fractions and has been applied into tropical forest soil stability research (Chen, 2012; Shang et al., 2013; Wang, 2010). Previous studies illustrated that the composition of the SOC chemical fractions is affected by soil microorganisms, soil properties, and carbon input (Elberling, Breuning-Madsen & Knicker, 2013; González-Pérez et al., 2008; Ktigel-Knabner, 1997; Lorenz, Lal & Jiménez, 2010). Topographic factors, such as elevation, slope, concavity and convexity, affect water flow and erosion, plants growth and litter decomposition, and hence influence SOC content and quality (Fernández-Romero, Lozano-García & Parras-Alcántara, 2014; Sun, Zhu & Guo, 2015). As such, there is inherent heterogeneity in SOC stability associated with the spatial variability of topographic factors. However, studies on factors influencing SOC stability have mainly focused on tree species, soil minerals protection or altitude (Angst et al., 2018) rather than topography.

Topography itself is a comprehensive factor, and it should be taken into consideration in SOC stability. Topography governs the allocation of water and heat resources, which affects the spatial allocation of vegetation and may thus affect the quantity and quality of SOC (Sun, Zhu & Guo, 2015). In addition, the movement of water and nutrients due to different topography types contributes to soil properties heterogeneity (Tsui, Chen & Hsieh, 2004). Studies found SOC fractions and stability varied across topography. Some studies found in lowland and plain areas alkyl carbon accounted for the most of the SOC fractions and indicated disturbance of soil and soil particle size be the key influencing factors (Chen, Xu & Mathers, 2004; Shang et al., 2013). However, other studies in plain and hills found a relative lower DI index (all less than 0.9) possibly due to the input of SOC and soil moisture (Lorenz, Lal & Jiménez, 2010; Wang, 2010). Studies in plateau found SOC that were not trapped in the iron nodules had a relative higher DI index (1.25) (Elberling, Breuning-Madsen & Knicker, 2013). We hypothesized that nutrients and soil environment factors would be the key influencing factors on SOC stability.

The Jianfengling montane rainforest is one of the best preserved tropical primaeval forests in China (Li et al., 2012). Previous studies have found that terrain heterogeneity in this area had a strong effect on the soil properties (Shi, 2012) and pointed out that SOC stability is significantly influenced by soil properties (Liu et al., 2007). Soil properties in different topographic positions also have distinctive characteristics under micro-climate conditions (Zhu et al., 2014), which fundamentally influences nutrients, hydrological processes, and thereby, the SOC stability across these topographies. However, there has been a systematic lack of studies specifically addressing how topographies influence the SOC stability by changing soil properties. The objectives of this study were to investigate the differences in soil properties and SOC stability at four topographic positions, and to identify the mechanisms of SOC stability in different topographies in tropical rainforests. To address these objectives, we collected soils from 60 quadrats in a 60ha dynamic plot in different topographical areas in the Jiangenling tropical montane rainforest. The stability of the SOC was demonstrated by SOC chemical fractions analysed by 13C-NMR.

## Materials and Methods

The study area was a 60 ha dynamics plot in the tropical montane rain forest (18.23°–18.50°N, 108.36°–109.05°E) with an elevation of 800–1,000 m, in Jianfengling, the southwest region of Hainan Island, China(Li & Zhou, 2002). The climate of the studied area is tropical and rainy climate, with a mean annual precipitation of 1,700–2,600 mm, and a mean annual temperature of 20–25 °C (Yang et al., 2016). The main plant families are Lauraceae, Rubiaceae, Fagaceae, Myrtaceae (Li & Zhou, 2002) and the soil belongs to the Ultisols order according to the USDA Soil Taxonomy.

**Figure 1:**
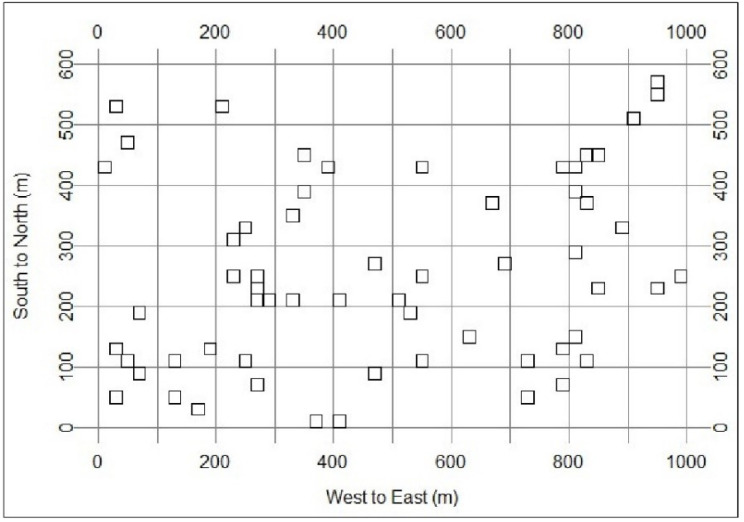
Distribution of soil sampling sites in the 60 ha plot in tropical forests.

Based on the technical specifications of the Centre for Tropical Forest Science, Smithsonian Institute, the 60 ha dynamics plot was divided into 1,500 quadrants, each 20 m × 20 m in size. Figure 1 shows the distribution of the 60 randomly selected soil sampling sites from the 1,500 quadrats. In each sampling site, the cutting ring method was used to obtain topsoil (0–10 cm) samples (each sample including four replicates). In total, 240 soil samples were collected and brought back to the laboratory. A part of each soil sample was stored at 4 °C to measure acid phosphatase activity (APA). A part was air-dried and sieved over 2 mm to characterize soil particle size (sand, silt and clay content). The rest of soil sample was air-dried and ground into 1 mm to analyse soil total nitrogen (TN), total phosphorus (TP), available phosphorus (AP), soil pH, and SOC chemical fractions.

Soil samples were pretreated with 10% (v/v) hydrofluoric acid (HF) solutions before solid-state 13C-CPMAS NMR analysis, to remove Fe^3+^ and Mn^2+^ in the soil, improving the signal/noise ratio. In detail, 10 g ground soil sample was vibrated for 2 h with 50 ml HF solutions and then centrifuged (3,000 rpm) for 10 min to separate the suspension. The above steps were repeated five times to obtain the precipitate (Mathers et al., 2002). The precipitate was washed 5 times with 50 ml deionized water to remove residual HF, then freeze-dried for further analysis. The fractions of SOC were measured with a 13C CPMAS/NMR, 4 mm probe pulse sequence. The CPTOSS 13C resonant frequency was 100.38 MHz, the 1 H resonant frequency was 399.16 MHz, rotation speed was 5 kHz, the contact time was 3 ms and cycle time was 1 s with a sampling frequency of 4 k. According to a previous study (Baldock et al., 1992), different peaks represent different C compounds: 0–45 ppm is alkyl carbon, 45–110 ppm is O-alkyl carbon, 110–160 ppm is aromatic carbon, and 160–220 ppm is carbonyl carbon. A total of 240 soil samples were analysed in the Analytical Centre of the Institute of Chemistry Chinese Academy of Sciences (Beijing City). MestRe-C software was used to analyse the chemical fractions of the SOC. We used two indices, the decomposition index (DI) and the inertia index (II), to represent SOC stability.

(1) \begin{eqnarray*}DI& = \frac{\text{alkyl carbon}}{\mathrm{O}-\text{alkyl carbon}\hspace*{2.22198pt}} \end{eqnarray*}

(2) \begin{eqnarray*}II& = \frac{\text{alkyl carbon}\hspace*{2.22198pt}+\text{aromatic carbon}}{\mathrm{o}-\text{alkyl carbon}\hspace*{2.22198pt}+\text{carboxyl carbon}} \end{eqnarray*}

The DI can reflect microbial processing with higher ratios indicating losses of more labile C relative to poorer-quality C compounds (Cusack et al., 2011), therefore, the higher ratio, the more stable is the SOC (Chen, 2012; Ostertag et al., 2008). Higher II values mean more alkyl carbon and aromatic carbon in the SOC, indicating higher SOC stability (Ostertag et al., 2008), which is beneficial for SOC accumulation.

Soil organic carbon and total N contents were determined by the K_2_Cr_2_O_7_–H_2_SO_4_ oxidation method and the Kjeldahl procedure respectively (Liu et al., 2012). Soil total phosphorus (TP) was extracted by the semimicro kelvin method and measured by an automatic flow analyzer (PROXIMA 1022/1/1, ALLIANCE instruments, France). Soil ammonium (NH_4_^+^-N, AN) content was extracted using 2 M KCl on an orbital shaker for 1 h under ambient temperature and then the suspension was filtered. The extracts were analysed by a continuous flow analytical system (SKALAR San++, SKALAR Co., Netherlands). Soil available phosphorus (AP) was determined by the ammonium chloride-hydrochloric acid extraction method (Bao, 2000), and acid phosphatase activity (APA) was determined by the phosphoric acid bisacid colourimetric method (Guan, 1986). Soil moisture (SM) was represented as the ratio of dry soil weight to soil water weight after fresh soil samples were dried at 105 °C for 24 h. Soil pH was detected by the potentiometric method, where 10 g air-dried soil was put into a 25 ml beaker and added with distilled water and kept for 30 min, after which the suspension’s pH value was measured with a corrected pH meter. Soil particle size distributions were determined by wet sieving (Chaudhari, Singh & Kundu, 2008). To investigate the effects of local topography on soil properties, we binned each data point into a topography type based on elevations, slopes and convexities by fuzzy *C*-mean clustering cluster analysis (Xu et al., 2015). Four topography types were identified: a flat area, a relative steep area, a steep area and a mountaintop area. We then performed one-way ANOVAs to test the differences in the soil properties among the topographic groups. The soil property was regarded as significantly different if *P*-value was ≤0.05. All results were shown as mean ± SE. Path analysis provides a way to examine the multiple relationships between SOC stability and environmental factors. Thus, a path analytic framework was applied to examine the influencing factors on SOC stability across the different topographies. As soil nutrients and other soil properties did not significantly differ among the four topographies. The flat and the top area were classified into the relative flat (RF) areas and the rest two types into the relative steep (RS) areas according to Wang et al. (2018). The RF areas had more favorable nutrients and environment than the RS areas and could be applied to validate the hypothesis we proposed previously.

All analyses were conducted using SPSS Version 20 and figures were generated by Origin (version 92 E).

## Results

### Soil properties in the different topography types

**Figure 2:**
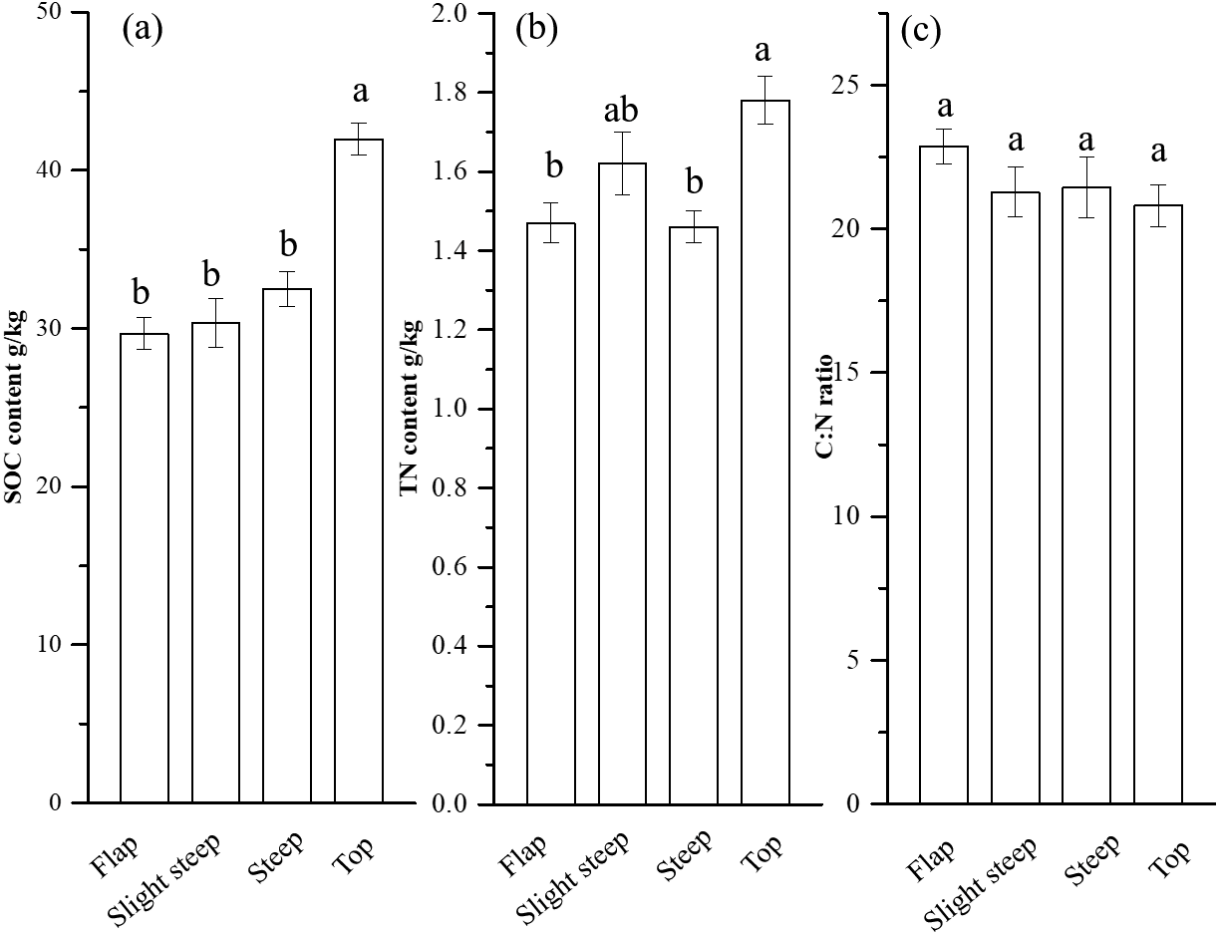
The changes of soil SOC (A), TN (B) and C:N ratio (C) among four topographies. The different lowercase letters indicate significant differences among topographies (*P* < 0.05).

Soil properties varied among the different topographies. To be specific, SOC increased from flat areas to mountaintop areas, with 29.66 g/kg in flat areas and 41.97 g/kg in mountaintop areas. Soil TN tended to increase in mountaintop areas, with 1.47 g/kg in flat areas and 1.78 g/kg in mountaintop areas. However, soil C:N ratios were similar across the different topographies. The AN, TP and SM were also the highest in the mountaintop areas, whereas the contents of pH was highest in the flat areas. Soil particle size did not change with topography except slit content was high in the flat area (Fig. 2 and Table 1).

### SOC components in the different topography types

**Figure 3:**
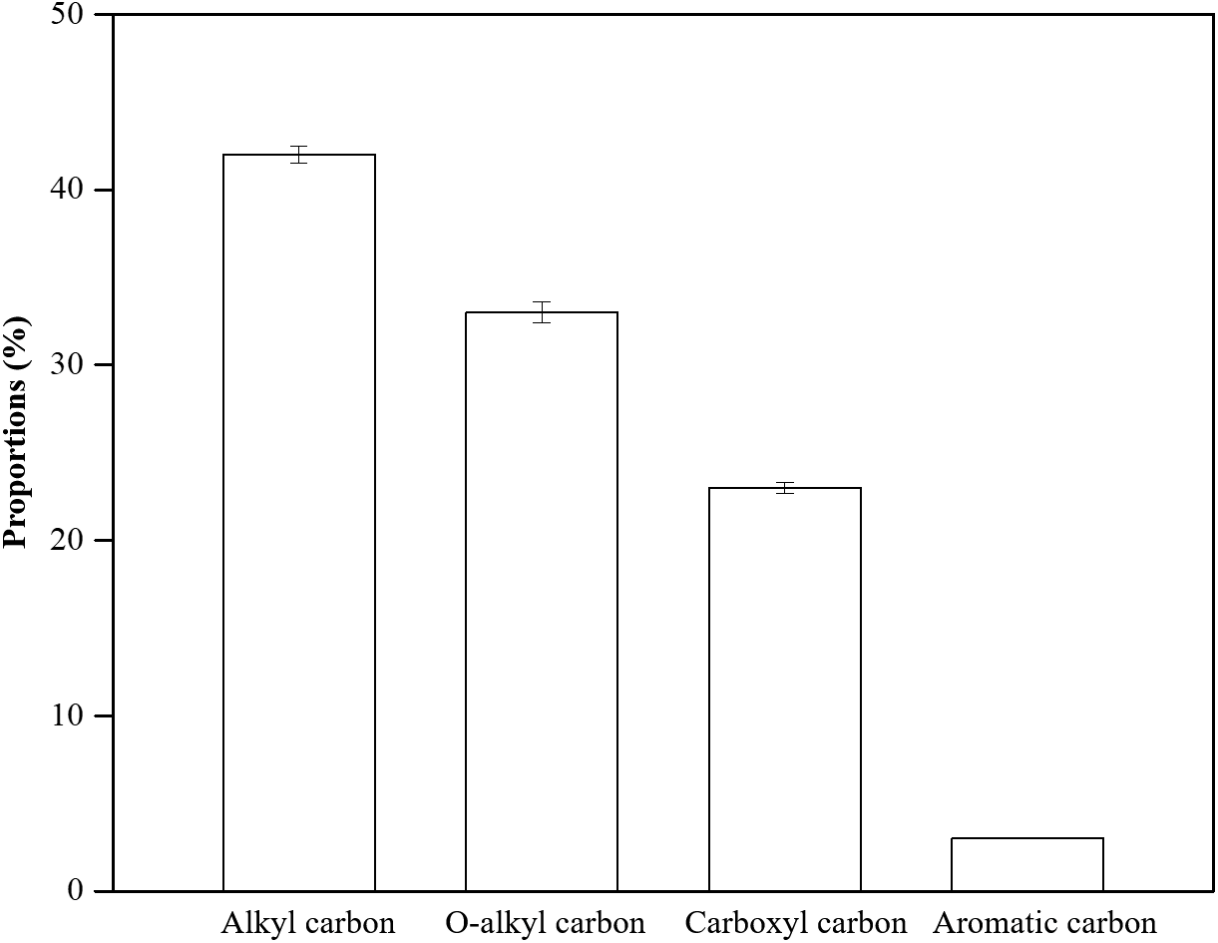
The distributions of SOC components in this tropical rainforest. Errors bars represent the standard error (SE).

**Figure 4:**
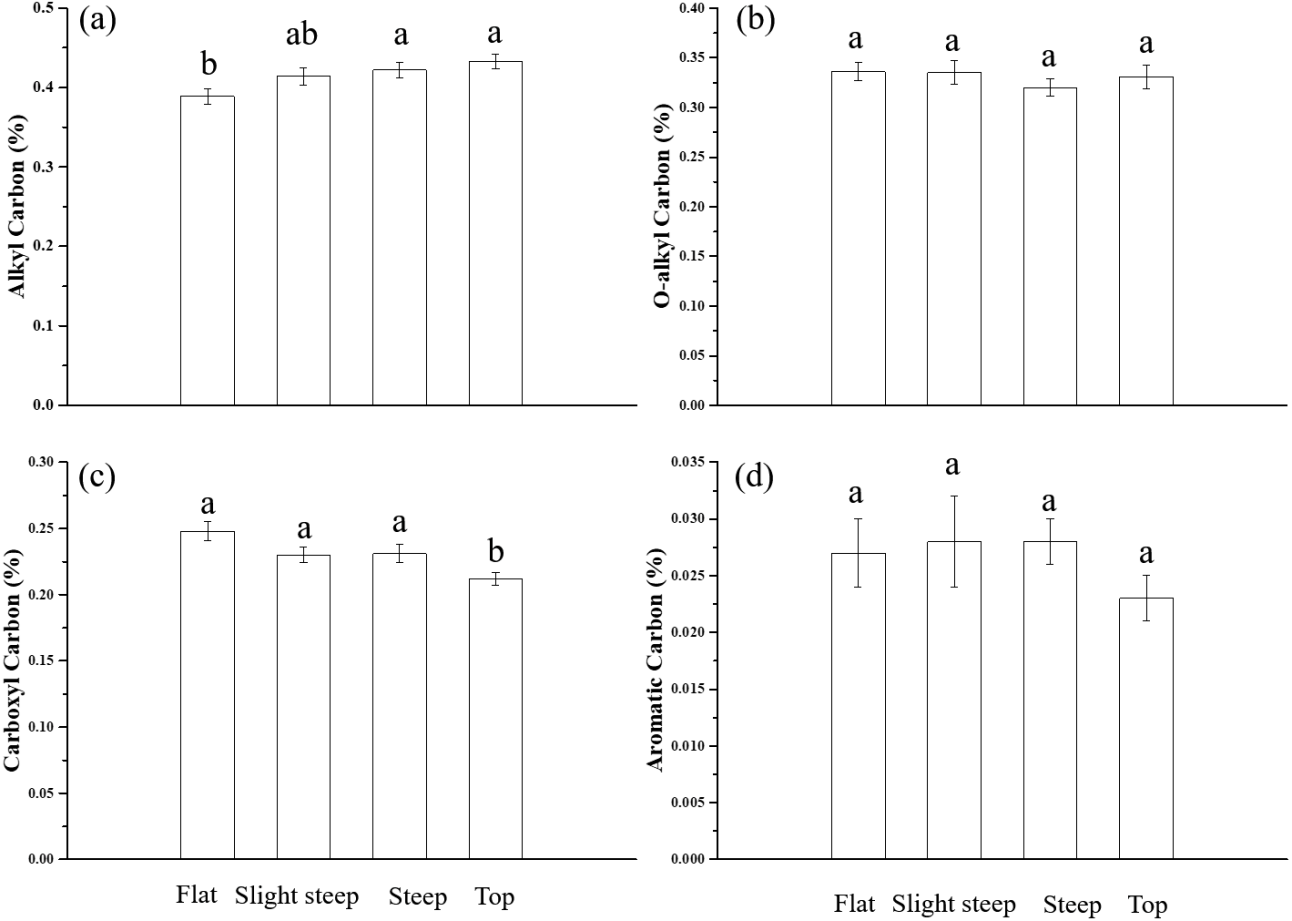
The comparisons of SOC fractions in the four topography types. (A) Alkyl carbon, (B) O-alkyl carbon, (C) carboxyl carbon, and (D) aromatic carbon. Error bars represent the standard error. The different lowercases mean significant differences among topographies (P < 0.05).

Alkyl carbon, on average 41.67% of the SOC, was the largest component of the SOC chemical fractions in the four different topography types (Fig. 3). Aromatic carbon, which accounted for only 2.61%, was the lowest SOC component. O-alkyl carbon and carboxyl carbon took up 33.08% and 22.80% of the SOC, respectively. As illustrated in Fig. 4, the overall distributions of the fractions of the SOC were similar across the different topography types. However, the proportion of alkyl carbon and carboxyl carbon was significantly altered by topography. The alkyl carbon content increased from the flat to mountaintop areas, with 38.87% in flat areas and 43.34% in mountaintop areas, while the carboxyl carbon content decreased from 24.78% in flat areas to 21.24% in mountaintop areas. O-alkyl carbon and aromatic carbon content did not change significantly with topography.

**Table 1:** Soil properties in different topographic areas (*n* = 60).

Variables	Flat (*n* = 12)	Slight steep (*n* = 16)	Steep (*n* = 13)	Top (*n* = 19)	Average (*n* = 60)
AN (mg/kg)	30.29 ± 6.04bc	42.16 ± 4.32ab	29.21 ± 5.69c	53.03 ± 3.41a	40.40 ± 2.62
TP (mg/kg)	74.12 ± 5.64b	82.50 ± 5.62ab	77.66 ± 5.07b	90.91 ± 3.68a	82.40 ± 2.54
AP (mg/kg)	2.71 ± 0.18a	3.03 ± 0.10a	2.92 ± 0.08a	2.30 ± 0.11a	2.93 ± 0.06
C:P ratio	468.65 ± 34.09a	433.61 ± 25.56a	442.74 ± 43.57a	407.08 ± 24.19a	434.20 ± 15.36
N:P ratio	20.56 ± 1.42a	20.26 ± 0.66a	20.39 ± 1.46a	19.68 ± 0.96a	20.16 ± 0.54
SM	20.88 ± 1.66cb	21.20 ± 1.52b	16.86 ± 0.83dc	25.28 ± 1.22a	21.49 ± 0.77
pH	4.42 ± 0.05a	4.32 ± 0.05b	4.31 ± 0.04ab	4.15 ± 0.02c	4.28 ± 0.02
Sand (%)	45.90 ± 4.81a	46.13 ± 3.28a	47.23 ± 2.72a	40.08 ± 2.20a	44.10 ± 1.61
Silt (%)	27.42 ± 3.57b	33.60 ± 2.22a	32.61 ± 2.85a	40.24 ± 2.51a	34.53 ± 1.51
Clay (%)	26.68 ± 6.58a	20.27 ± 4.14a	20.16 ± 4.13a	19.68 ± 3.18a	21.37 ± 2.17

**Notes.**

Abbreviation AN was ammoniacal nitrogen TP was total phosphorus AP was available phosphorus C:P ratio was calculated as SOC/ TP N:P ratio was calculated as TN/TP SM was soil moisture n was sample size

All values were expressed as mean values ± SE. The different lowercases mean significant differences among topographies (*P* < 0.05).

### SOC stability indices in the different topography types

**Figure 5:**
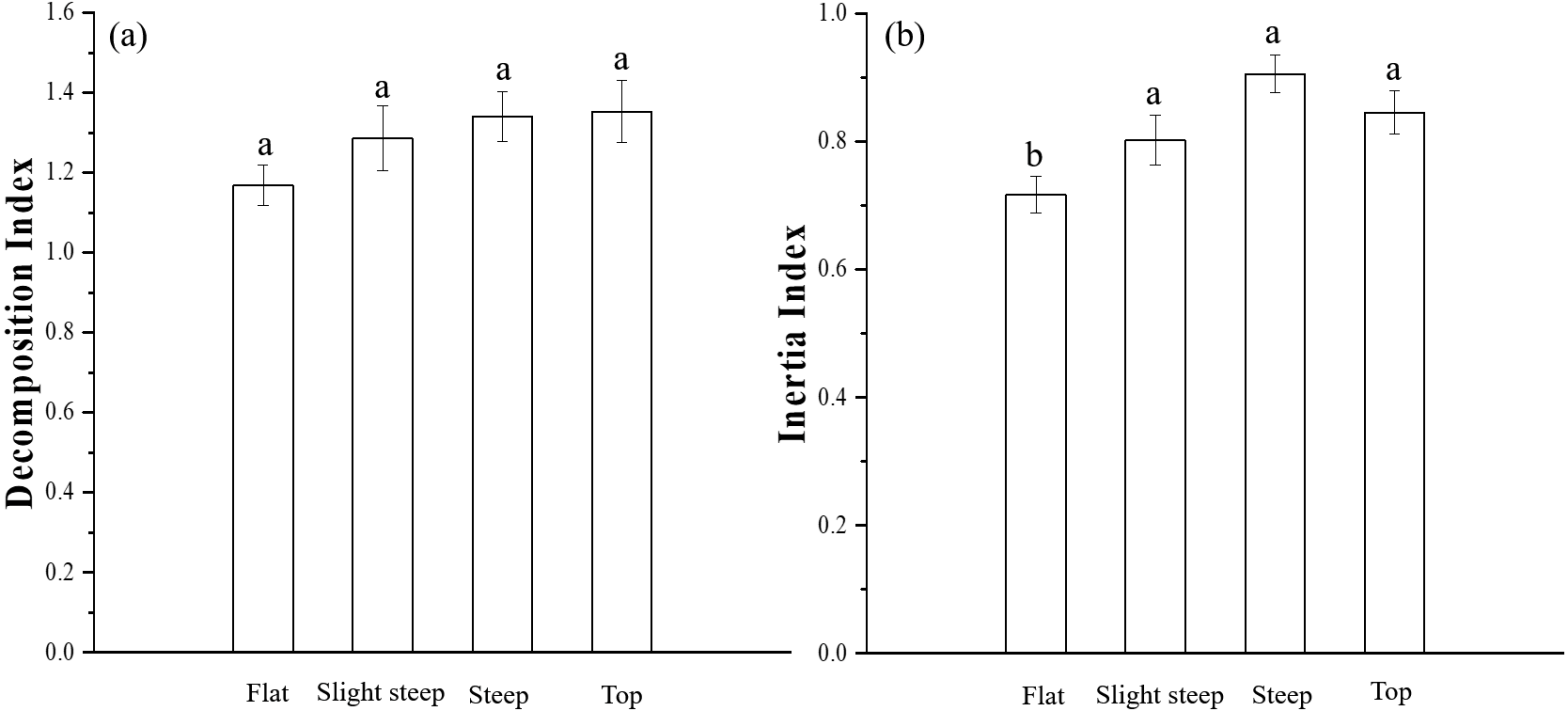
Difference of decomposition index (DI) and inertia index (II) among topographies. (A) decomposition index, (B) inertia index. Error bars represent the standard error. The same letter means variables in the different topography types are not significantly different.

In all four topographies, the DI was higher than 1 and the II was higher than 0.7. The DI was not significantly different among the topographies, while II was significantly different in different topographies with the lowest II value in the flat areas (0.72) and the highest value in the steep areas (0.91) (Fig. 5).

### Influencing factors on SOC stability in the different topography types

**Figure 6:**
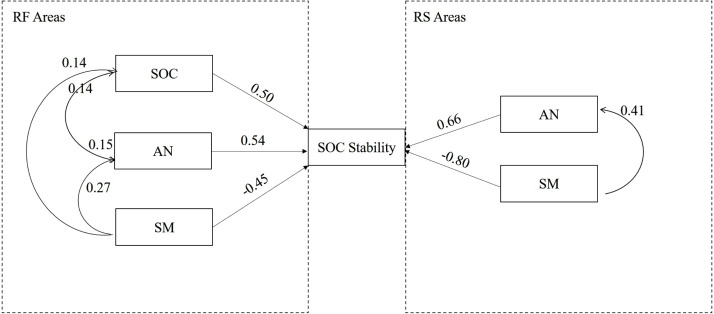
Influencing factors and mechanisms of SOC stability in two topographies. Values aside the straight line are direct path coefficient, and values aside the curve are indirect path coefficient. The direction of arrow indicates the effect direction.

**Figure 7:**
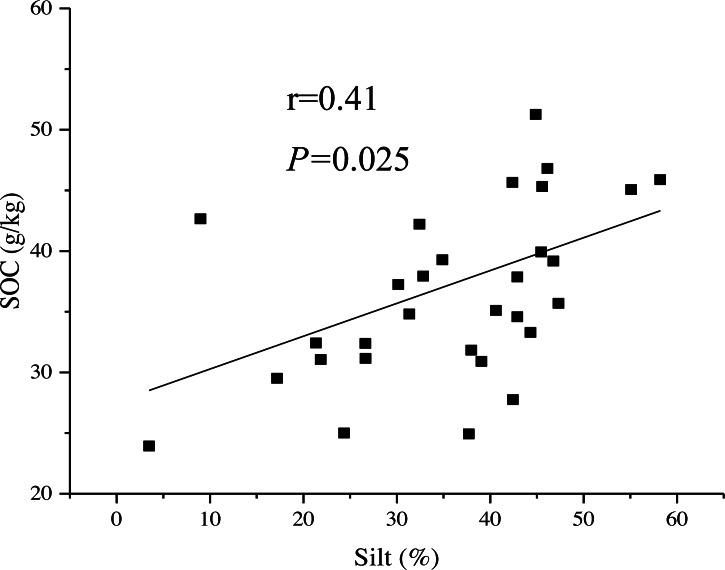
The simple liner relationship between soil texture and SOC content in the relative flat areas.

Figure 6 shows that the influencing factors and mechanisms of SOC stability were different in the different topography types. In the RF areas, SOC, AN and SM together explained 52% of the variability in the SOC stability. SOC and AN directly positively affected SOC stability; high SOC and AN strengthened SOC stability. SM directly negatively influenced SOC stability, however, the direct negative effect of SM was partly offset by the indirect positive effect of SM on AN and SOC, thus, SM had little overall effects on SOC stability. Besides, SOC were positively correlated with soil silt content in this area (Fig. 7). However, soil particle size was not adopted by the model.

In the RF areas, SM and AN together explained 26% variability of SOC stability. In the RS areas, SM and AN also affected SOC stability by 42%. The effect was the same as for RF areas, though the effect of SM was larger than that of AN on SOC stability. Increasing SM decreased SOC stability, though this negative effect was partly offset bySM’s indirect effect on AN. AN directly positively affected SOC stability.

Soil AN and SM were influencing factors on SOC stability in the RF areas and the RS areas, however, the extent of their effect differed. Specifically, SM was a negligible limiting factor in the RF areas, whereas SM was the most important limiting factor in the RS areas. The path analyses model had an R square of 0.53 in the RF areas, while this was 0.42 in the RS areas (Table S1).

## Discussion

The chemical fractions of the SOC in the different topographic areas were characterized by alkyl carbon > O-alkyl carbon > carboxyl carbon > aromatic carbon. The ranking of the SOC fractions is similar to those of subtropical evergreen board-leaved forest and Masson pine in China (Shang et al., 2013), and subtropical natural forest and hoop pine plantation in Australia (Chen, Xu & Mathers, 2004) with 13CNMR technology. Alkyl carbon was the largest component of the SOC fractions, indicating that the SOC comprised a higher percentage of passive carbon in SOC. The DI (alkyl carbon/O-alkyl carbon) for the 60 ha was 1.29, which is higher than moist semi-deciduous tropical forest soils in Ghana (1.25) (Elberling, Breuning-Madsen & Knicker, 2013), Costa Rican old live oak forest (0.87) and old-growth dry tropical forests (0.70) (Lorenz, Lal & Jiménez, 2010), plantations in the subtropics of China (Pinus massoniana, Castanopsis hystrix, Michelia macclurei, Mytilaria laosensis, all < 0.8) (Wang, 2010), evergreen broad-leaved forest (0.76), Cunninghamia lanceolata forest (0.89), Cryptomeria fortune forest (0.78), and coniferous and broad-leaved mixed forest (0.53) in the subtropics of China (Zhang et al., 2015). The DI of all the different topography types was all higher than 1, indicating a rather stable SOC in the tropical montane rainforest of Jiangfengling, Hainan Island (González-Pérez et al., 2008). Studies of changes in SOC and its fractions of forest along a climatic gradient in China found sites with higher mean annual temperature would have greater alkyl C proportion and higher DI value as active SOC decomposition processes accelerated (Sun et al., 2019). Path analysis showed that the SOC contents only affected SOC stability in RF areas, while AN and SM affected SOC stability in both topographies. Studies have found that interactions between SOC and mineral surfaces can stabilise SOC, whereby stable organic-mineral bonds were formed through anion and inner-sphere ligand-exchange reaction (Wiesmeier et al., 2019). Tropical forest with highly weathered soils contains high concentrations of iron (Sanchez, 1976) that could protect C from microbes and enhance SOC stability. Studies found Fe^3+^ was positively correlated with the SOC content (Wang et al., 2019). The SOC affected SOC stability in the RF areas might be due to the fact that SOC content in the RF areas was significantly higher than that in the RS areas (37.21 > 31.32 g/kg, *p* < 0.01).

SOC stability depends not only on its chemical characteristics, but also on the soil microenvironment (Yang et al., 2020). Soil biotic community and the soil microenvironment are key factors that affecting the SOC stability (Schmidt et al., 2011; Yang et al., 2018). Soil N, especially in the bioavailable forms, such as ammonium is an essential component of all living organisms. Soil N dynamics are mainly driven by microbes (Levy-Booth, Prescott & Grayston, 2014) and microbes mainly assimilate of AN (Cheng et al., 2015), however, some studies have found that nitrogen addition negatively affects soil microbial growth, composition and function (Chen et al., 2018). The soil AN content in the study area was rather high at about 40.4 mg/kg while the value in Amazon pristine forest was 5.7 mg/kg (Hamaoui et al., 2016) and the value in a secondary tropical forest in Southern China was 2.12 mg/kg (Wang et al., 2014). Therefore, microorganism activities and functions might be suppressed by the high AN content in this area, resulting in a slow decomposition rates and consequently high SOC stability in this tropical forest.

Soil moisture is a key factor influencing SOC mineralization in terrestrial ecosystems (Liu, Zhang & Wan, 2009; Moyano, Manzoni & Chenu, 2013). Soil moisture can affect SOC decomposition directly or indirectly. Topography influences soils by the transport of fine soil particle towards the base of slopes and lower slope positions in what are called depositional areas, which tend to have high organic materials and water-holding capacity (Chapin III, Matson & Vitousek, 2012). The above process results in the deposition of more active carbon than passive carbon, explaining the low DI and II in the RF areas. This explains why SM was a negative influencing factor of SOC stability in the RS and RF areas (Fig. 6). Moreover, the SOC in the flap areas was the most unstable among the four topography types (Fig. 4). In terms of indirect effects, decomposition of soil organic matter depends on factors such as soil mineralogy, redox potential and electron acceptor availability, which are controlled by soil water regimes (Ro, Ji & Lee, 2018). Studies have found that an increase in soil water content benefits dissolved organic carbon dissolution and nutrients transfer, which stimulates the activity of microorganisms involved in organic carbon decomposition (Goebel et al., 2007). Higher SM results in higher rates of substate supply to the microbes and, thereby, higher microbial growth. In addition, soil moisture availability can affect the vegetation distribution and structure (D’Odorico et al., 2007; Ruiz-Sinoga et al., 2011), affecting the amount and quality of litter, which in turns influences the soil organic matter decomposition rate (Rodríguez-Iturbe & Porporato, 2007). On the other hand, AN is water-soluble nitrogen which is vulnerable to the changes of soil water. Hence, SM could negatively affect SOC stability by positively influencing the soil microbes and AN content.

## Conclusions

Our analyses of the effects of small-scale topography on SOC stability in a tropical mountain rain forest showed that topographic heterogeneity altered SOC stability by changing soil nutrients and soil moisture. Our findings indicated that SOC is relatively stable in the tropical montane rainforest topsoil and AN and SM were the main influencing factors of SOC stability both in RF and RS areas. Furthermore, SOC and AN positively promote SOC stability while SM was a limiting factor on SOC stability in this study areas. Accordingly, soil nutrients were the key influencing factors in RF areas but not in RS areas. Our hypothesis that nutrients and soil environment were the key influencing factor in studied areas were partly confirmed. The path analysis models explained less than 50% of the SOC stability in the RS areas, thus the mechnism remains to be further explored.

## Supplemental Information

10.7717/peerj.12057/supp-1 Supplemental Information 1 Raw data

10.7717/peerj.12057/supp-2 Supplemental Information 2 The parameters of path analysis for SOC stability in the two different topography types

## Additional Information and Declarations

### Data Availability

The following information was supplied regarding data availability: The raw measurements are available in the Supplemental File.

## References

[ref-1] Angst G, Mueller KE, Eissenstat DM, Trumbore S, Freeman KH, Hobbie SE, Chorover J, Oleksyn J, Reich PB, Mueller CW (2018). Soil organic carbon stability in forests: distinct effects of tree species identity and traits. Global Change Biology.

[ref-2] Baldock JA, Oades JM, Waters AG, Peng X, Vassallo AM, Wilson MA (1992). Aspects of the chemical structure of soil organic materials as revealed by solid-state 13C NMR spectroscopy. Biogeochemistry.

[ref-3] Bao S (2000). Soil agrochemical analysis.

[ref-4] Chapin III FS, Matson PA, Vitousek PM (2012). Principles of terrestrial ecosystem ecology.

[ref-5] Chaudhari SK, Singh R, Kundu DK (2008). Rapid textural analysis for saline and alkaline soils with different physical and chemical properties. Soil Science Society of America Journal.

[ref-6] Chen CR, Xu ZH, Mathers NJ (2004). Soil carbon pools in adjacent natural and plantation forests of subtropical Australia. Soil Science Society of America Journal.

[ref-7] Chen X (2012). The effects of major environmental changes on soil organic carbon fractions in subtropical forests. D. Sc. Thesis.

[ref-8] Chen X, Deng Q, Lin G, Lin M, Wei H (2018). Changing rainfall frequency affects soil organic carbon concentrations by altering non-labile soil organic carbon concentrations in a tropical monsoon forest. Science of the Total Environment.

[ref-9] Christensen B (1996). Carbon in primary and secondary organomineral complexes. Chapter, structure and organic matter storage in agricultural soils.

[ref-10] Cusack D, Silver W, Torn M, Burton S, Firestone M (2011). Changes in microbial community characteristics and soil organic matter with nitrogen additions in two tropical forests. Ecology.

[ref-11] D’Odorico P, Caylor K, Okin GS, Scanlon TM (2007). On soil moisture-vegetation feedbacks and their possible effects on the dynamics of dryland ecosystems. Journal of Geophysical Research: Biogeosciences.

[ref-12] Elberling B, Breuning-Madsen H, Knicker H (2013). Carbon sequestration in iron-nodules in moist semi-deciduous tropical forest soil. Geoderma.

[ref-13] Fernández-Romero ML, Lozano-García B, Parras-Alcántara L (2014). Topography and land use change effects on the soil organic carbon stock of forest soils in Mediterranean natural areas. Agriculture, Ecosystems & Environment.

[ref-14] Fontaine S, Barot S, Barre P, Bdioui N, Mary B, Rumpel C (2007). Stability of organic carbon in deep soil layers controlled by fresh carbon supply. Nature.

[ref-15] Goebel M-O, Woche SK, Bachmann J, Lamparter A, Fischer WR (2007). Significance of wettability-induced changes in microscopic water distribution for soil organic matter decomposition. Soil Science Society of America Journal.

[ref-16] González-Pérez M, Torrado PVidal, Colnago LA, Martin-Neto L, Otero XL, Milori DMBP, Gomes FH (2008). 13C NMR and FTIR spectroscopy characterization of humic acids in spodosols under tropical rain forest in southeastern Brazil. Geoderma.

[ref-17] Guan S (1986). Soil enzyme and research methods.

[ref-18] Hamaoui GS, Rodrigues JLM, Bohannan BJM, Tiedje JM, Nüsslein K (2016). Land-use change drives abundance and community structure alterations of thaumarchaeal ammonia oxidizers in tropical rainforest soils in Rondônia, Brazil. Applied Soil Ecology.

[ref-19] Hassink J (1997). The capacity of soils to preserve organic C and N by their association with clay and silt particles. Plant and Soil.

[ref-20] Ktigel-Knabner I (1997). 13C and 15N NMR spectroscopy as a tool in soil organic matter studies. Geoderma.

[ref-21] Levy-Booth DJ, Prescott CE, Grayston SJ (2014). Microbial functional genes involved in nitrogen fixation, nitrification and denitrification in forest ecosystems. Soil Biology and Biochemistry.

[ref-22] Li Y, Xu H, Luo T, Chen D, Lin M (2012). Permanent monitoring and research dataset of Chinese ecosystem:forest ecosystem, Jianfengling Station.Bio-species checklist.

[ref-23] Li Y, Zhou G (2002). Research and conservation of tropical forest and the biodiversity, Hainan Island.

[ref-24] Liu M, Chen X, Guo J, Li H, Hu F (2007). Soil biota on soil organic carbon stabilization. Advances in Earth Science.

[ref-25] Liu W, Chen S, Qin X, Baumann F, Scholten T, Zhou Z, Sun W, Zhang T, Ren J, Qin D (2012). Storage, patterns, and control of soil organic carbon and nitrogen in the northeastern margin of the Qinghai–Tibetan Plateau. Environmental Research Letters.

[ref-26] Liu W, Zhang Z, Wan S (2009). Predominant role of water in regulating soil and microbial respiration and their responses to climate change in a semiarid grassland. Global Change Biology.

[ref-27] Lorenz K, Lal R, Jiménez JJ (2010). Characterization of soil organic matter and black carbon in dry tropical forests of Costa Rica. Geoderma.

[ref-28] Mathers NJ, Xu Z, Berners-Price SJ, Perera MCS, Saffigna PG (2002). Hydrofluoric acid pre-treatment for improving 13C CPMAS NMR spectral quality of forest soils in south-east Queensland, Australia. Australian Journal of Soil Research.

[ref-29] Moyano FE, Manzoni S, Chenu C (2013). Responses of soil heterotrophic respiration to moisture availability: an exploration of processes and models. Soil Biology and Biochemistry.

[ref-30] Ostertag R, Marín-Spiotta E, Silver WL, Schulten J (2008). Litterfall and decomposition in relation to soil carbon pools along a secondary forest chronosequence in Puerto Rico. Ecosystems.

[ref-31] Pan Y, Birdsey RA, Fang J, Houghton R, Kauppi PE, Kurz WA, Phillips OL, Shvidenko A, Lewis SL, Canadell JG, Ciais P, Jackson RB, Pacala SW, McGuire AD, Piao S, Rautiainen A, Sitch S, Hayes D (2011). A large and persistent carbon sink in the world’s forests. Science.

[ref-32] Ro H-M, Ji Y, Lee B (2018). Interactive effect of soil moisture and temperature regimes on the dynamics of soil organic carbon decomposition in a subarctic tundra soil. Geosciences Journal.

[ref-33] Rodríguez-Iturbe I, Porporato A (2007). Ecohydrology of water-controlled ecosystems: soil moisture and plant dynamics.

[ref-34] Ruiz-Sinoga JD, Gabarrón Galeote MA, Martinez Murillo JF, Garcia Marín R (2011). Vegetation strategies for soil water consumption along a pluviometric gradient in southern Spain. Catena.

[ref-35] Sanchez P (1976). Properties and management of soils in the tropics.

[ref-36] Schmidt MW, Torn MS, Abiven S, Dittmar T, Guggenberger G, Janssens IA, Kleber M, Kogel-Knabner I, Lehmann J, Manning DA, Nannipieri P, Rasse DP, Weiner S, Trumbore SE (2011). Persistence of soil organic matter as an ecosystem property. Nature.

[ref-37] Shang S, Jiang P, Song Z, Li Y, Lin L (2013). Composition and stability of organic carbon in the top soil under different forest types in subtropical China. Acta Ecologica Sinica.

[ref-38] Shi L (2012). Study on the spatial heterogeneity of soil physical and chemical properties of primary tropical montane rainforest. M. Ecology. Thesis.

[ref-39] Six J, Conant RT, Paul EA, Paustian K (2002). Stabilization mechanisms of soil organic matter: Implications for C-saturation of soils. Plant and Soil.

[ref-40] Stockmann U, Adams MA, Crawford JW, Field DJ, Henakaarchchi N, Jenkins M, Minasny B, McBratney AB, VdRd Courcelles, Singh K, Wheeler I, Abbott L, Angers DA, Baldock J, Bird M, Brookes PC, Chenu C, Jastrow JD, Lal R, Lehmann J, O’Donnell AG, Parton WJ, Whitehead D, Zimmermann M (2013). The knowns, known unknowns and unknowns of sequestration of soil organic carbon. Agriculture, Ecosystems & Environment.

[ref-41] Sun X, Tang Z, Ryan MG, You Y, Sun OJ (2019). Changes in soil organic carbon contents and fractionations of forests along a climatic gradient in China. Forest Ecosystems.

[ref-42] Sun W, Zhu H, Guo S (2015). Soil organic carbon as a function of land use and topography on the Loess Plateau of China. Ecological Engineering.

[ref-43] Tsui C-C, Chen Z-S, Hsieh C-F (2004). Relationships between soil properties and slope position in a lowland rain forest of southern Taiwan. Geoderma.

[ref-44] Wang H (2010). Soil carbon sequestration and the related processes in four subtropical plantations in southern China Doctor.

[ref-45] Wang J, Han X, Yide L, Mingxian L, Zhang Z, Tushou L, Dexiang C (2018). Effects of topographic heterogeneity on community structure and diversity of woody plants in Jianfengling tropical montane rainforest. Scientia Silvae Sinicae.

[ref-46] Wang F, Li J, Wang X, Zhang W, Zou B, Neher DA, Li Z (2014). Nitrogen and phosphorus addition impact soil N(2)O emission in a secondary tropical forest of South China. Scientific Reports.

[ref-47] Wang P, Wang J, Zhang H, Dong Y, Zhang Y (2019). The role of iron oxides in the preservation of soil organic matter under long-term fertilization. Journal of Soils and Sediments.

[ref-48] Wiesmeier M, Schad P, Von Lützow M, Poeplau C, Spörlein P, Geuß U, Hangen E, Reischl A, Schilling B, Kögel-Knabner I (2014). Quantification of functional soil organic carbon pools for major soil units and land uses in southeast Germany (Bavaria). Agriculture, Ecosystems & Environment.

[ref-49] Wiesmeier M, Urbanski L, Hobley E, Lang B, Von Lützow M, Marin-Spiotta E, Van Wesemael B, Rabot E, Ließ M, Garcia-Franco N, Wollschläger U, Vogel H-J, Kögel-Knabner I (2019). Soil organic carbon storage as a key function of soils - A review of drivers and indicators at various scales. Geoderma.

[ref-50] Xu H, Li Y, Lin M, Wu J, Luo T, Zhou Z, Chen D, Yang H, Li G, Liu S (2015). Community characteristics of a 60 ha dynamics plot in the tropical montane rain forest in Jianfengling, Hainan Island. Biodiversity Science.

[ref-51] Yang J, Li G, Qian Y, Yang Y, Zhang F (2018). Microbial functional gene patterns related to soil greenhouse gas emissions in oil contaminated areas. Science of the Total Environment.

[ref-52] Yang H, Li Y, Ren H, Luo T, Chen R, Liu W, Chen D, Cu H, Zhou Z, Lin M, Yang Q, Yao H, Zhou G (2016). Soil organic carbon density and influencing factors in tropical virgin forests of Hainan Island, China. Chinese Journal of Plant Ecology.

[ref-53] Yang J, Li A, Yang Y, Li G, Zhang F (2020). Soil organic carbon stability under natural and anthropogenic-induced perturbations. Earth-Science Reviews.

[ref-54] Zhang Y, Hu H, Huang Y, li T (2015). Effects of different vegetation restoration models on molecular structure and stability of soil organic carbon. Research of Environmental Sciences.

[ref-55] Zhu H, Wu J, Guo S, Huang D, Zhu Q, Ge T, Lei T (2014). Land use and topographic position control soil organic C and N accumulation in eroded hilly watershed of the Loess Plateau. Catena.

