# Supplementary material for: The stability of soil organic carbon across topographies in a tropical rainforest
Source: PeerJ. 2021 Aug 27;9:e12057. doi: 10.7717/peerj.12057 (PMC8404569; doi:10.7717/peerj.12057)
Supplement: Supplemental Information 2 [file peerj-09-12057-s002.docx]

Table S1. The parameters of path analysis for SOC stability in the two different topography types

| **Topography type** | **Factors** | **SCC** | **DPC** | **SOC** | **AN** | **SM** | **Decision coefficient** | **Determination coefficient** | **R square of the model** |
| --- | --- | --- | --- | --- | --- | --- | --- | --- | --- |
| Relative flat areas | SOC | 0.52 | 0.50 | - | 0.15 | -0.13 | 0.27 | 0.26 | 0.53 |
|  | AN | 0.45 | 0.54 | 0.14 | - | -0.23 | 0.19 | 0.24 |  |
|  | SM | -0.05 | -0.45 | 0.14 | 0.27 | - | -0.16 | 0.02 |  |
| Relative Steep areas | SM | -0.40 | -0.80 |  | 0.41 |  | 0.00 | 0.32 | 0.42 |
|  | AN | 0.16 | 0.66 |  |  | -0.50 | -0.22 | 0.11 |  |
